# Correlation between Sperm Micro Ribonucleic Acid-34b and -34c Levels and Clinical Outcomes of Intracytoplasmic Sperm Injection in Men with Male Factor Infertility

**DOI:** 10.3390/ijms232012381

**Published:** 2022-10-16

**Authors:** Ling-Yu Yeh, Robert Kuo-Kuang Lee, Ming-Huei Lin, Chih-Hung Huang, Sheng-Hsiang Li

**Affiliations:** 1 Institute of Biochemical and Biomedical Engineering, Department of Chemical Engineering & Biotechnology, National Taipei University of Technology, Taipei 106, Taiwan; 2 Department of Medical Research, MacKay Memorial Hospital, Tamsui District, New Taipei 251, Taiwan; 3 MacKay Junior College of Medicine, Nursing, and Management, Taipei 112, Taiwan; 4 Department of Obstetrics and Gynecology, MacKay Memorial Hospital, Taipei 104, Taiwan; 5 Department of Chemical Engineering & Biotechnology, National Taipei University of Technology, Taipei 106, Taiwan

**Keywords:** sperm, microRNA, intracytoplasmic sperm injection, teratozoospermia, asthenozoospermia, male factor infertility

## Abstract

Few studies have examined the correlation between sperm miRNA levels and clinical outcomes of intracytoplasmic sperm injection (ICSI). In this study, we aimed to assess the correlation of sperm miR-34b, miR-34c, miR-122, and miR-429 levels with ICSI outcomes in men with teratozoospermia and asthenozoospermia. TaqMan microRNA quantitative polymerase chain reaction was used to evaluate the relative expression of miRNAs in sperm. The relative miRNA levels quantified using a comparative method found that the four miRNAs were not associated with fertilization rate and early embryo development. However, revels of miR-34b and miR-34c in teratozoospermia sperm of the live birth group were significantly higher than those in the non-live birth group. Receiver operating characteristic curve analysis revealed that the optimal cut-off delta cycle threshold values of miR-34b and miR-34c were 8.630 and 7.883, respectively. Statistical analysis found that the levels of miR-34b and the miR-34c in teratozoospermic and asthenozoospermic sperm above the thresholds were not associated with the fertilization rate and the high-quality embryo rate above 50%; however, they were more likely to exhibit higher implantation, pregnancy, and live birth rates. miR-34b and miR-34c were significantly associated with ICSI clinical outcomes in male factor infertility, especially teratozoospermia. Further validation is required before it becomes a clinically valid reference indicator.

## 1. Introduction

MicroRNAs (miRNAs) are a class of small non-coding RNAs that regulate post-transcriptional repression by pairing to the 3’-untranslated regions of messenger RNAs (mRNAs) of protein-coding genes [1], participate in various physiological responses, and affect cell growth, development, meiosis, apoptosis, differentiation, and other processes [2]. They are widely present in male testis, seminal plasma, and sperm [3] and play an essential role in regulating genes involved in spermatogenesis [4].

Studies have found that miRNA expression in the male reproductive system is associated with infertility [5,6,7,8]. Human miR-34 and miR-449 family miRNA levels are reduced in human testes with abnormal spermatogenesis. Bioinformatics analysis showed five miRNAs (hsa-mir-34b*, hsa-mir-34b, hsa-mir-34c-5p, hsa-mir-449a, and hsa-mir-449b*) are involved in spermatogenesis and target specific genes associated with apoptosis, cell proliferation, and differentiation [5]. Dysregulation of miR-34b, miR-34c-5p, miR-429, and miR-122 was found in purified human sperm and testicular tissue from subfertile and non-obstructive azoospermia (NOA) males [6]. Seven miRNAs, including miR-34c-5p, miR-122, miR-146b-5p, miR-181a, miR-374b, miR-509-5p, and miR-513a-5p, were identified as altered expression profile in the seminal plasma of infertile men [7]. In addition, seminal plasma miR-34, miR-122, and miR-509 are down-regulated in NOA and oligozoospermia patients compared with fertile controls [8].

miR-34 is a conserved miRNA family whose members, including miR-34a, miR-34b, and miR-34c [9], regulate cellular senescence, apoptosis, and cell cycle [10,11,12]. miR-34b and miR-34c are expressed in male gonads [13,14,15]. The target genes of the miR-34 family correspond to many cell cycle regulators. In rhesus monkey testes, the predicted target gene NOTCH1 of miR-34b and miR-34c is a crucial regulator of germ cell differentiation and survival [16]. In addition, the DAZL gene, critical for mouse germ cell differentiation, may also be a target gene of miR-34b and miR-34c [17]. miR-34c directly targets *TGIF2* and *NOTCH2* and has been shown to play an essential role in spermatogenesis in male germ cells [18]. miR-34b and miR-34c deficiency impair meiosis and the late stages of spermatogenesis, resulting in oligoasthenoteratozoospermia in mice [19]. Furthermore, miR-34c has been shown to play an essential role in the first cleavage division of mouse zygotes [20]. It has also been correlated with clinical outcomes in patients with intracytoplasmic sperm injection (ICSI) [21] and in vitro fertilization (IVF) [22].

miR-122, primarily expressed in polysomes of late-stage male mouse germ cells, is involved in the post-transcriptional regulation of mRNAs in testis [23] and chromatin remodeling during spermatogenesis [24]. miR-122 expression is associated with abnormal sperm development and mediates suppression of protein expression associated with sperm development [24]. Its levels are upregulated in the semen of infertile males with semen abnormalities [25] compared with the normal semen of healthy males. Its expression is significantly increased in the sperm of men with severe and moderate oligoasthenoteratozoospermia compared with normozoospermia [26]. However, miR-122 levels are lower in the sperm of oligozoospermic and asthenozoospermic men than in normozoospermic men [27,28]. These results appear to be somewhat conflicting and require further study. Furthermore, in bovine sperm, miR-122 expression is elevated in low motile sperm [29].

Sperm miR-429 levels are upregulated in asthenozoospermic men, and it may regulate sperm motility and viability by inhibiting the expression of heat shock protein A4L [30]. In addition, the expression of miR-429 in sperm and testicular tissues of azoospermic patients is higher than that of normal controls [6]. miR-429 level in seminal plasma is significantly increased in azoospermic men compared with normozoospermia males [31]. It is also elevated in spermatozoa and seminal plasma of oligozoospermia and asthenozoospermia males [27].

Although there have been many studies on the functional roles of specific miRNAs during spermatogenesis and their expression levels in abnormal spermatozoa, the association between sperm miRNAs and artificial reproductive technology treatment and clinical outcomes after embryo implantation in patients has not been widely studied. This study aimed to investigate the correlation between the expression levels of four crucial miRNAs, miR-34 b/c, miR-122, and miR-429, in spermatozoa and clinical outcomes using ICSI patient samples.

## 2. Results

### 2.1. Physiological Data Analysis of Subjects

**Figure 1:**
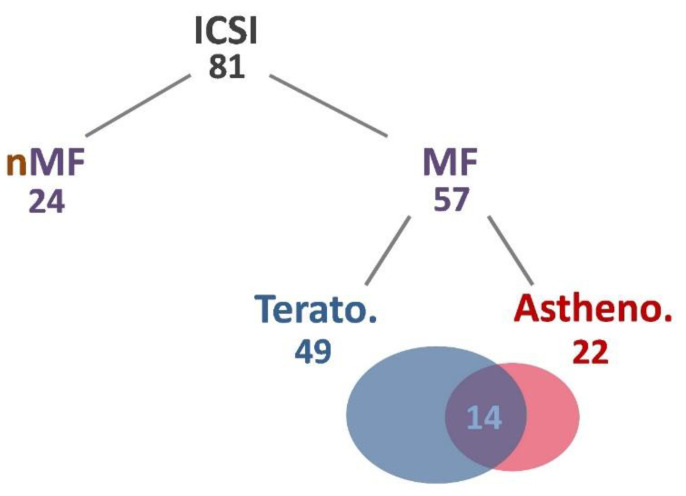
Distribution of sperm samples. Numbers indicate the number of cases. Abbreviations: Astheno, asthenozoospermia; ICSI, intracytoplasmic sperm injection; MF, male factor; nMF, non-male factor; Terato, teratozoospermia.

This study included 81 couples receiving ICSI treatments (81 cycles, 1 cycle for each pair). Male patients were between 27–55 years old, with an average age of 39.11, and their wives were between 27–46 years old, with an average age was 36.94. The average infertility period was 3.48 years. Of the 81 ICSI specimens, 57 were male factor infertility, including 49 teratozoospermia and 22 asthenozoospermia, of which 14 were both teratozoospermia and asthenozoospermia. An additional 24 samples entered the course of treatment not due to male factors, including poor fertilization rates in the previous cycle, egg freezing and thawing for fertilization, and some older patients with unexplained infertility (Figure 1). Oligozoospermia has screened out this classification because there was insufficient sperm for analysis.

Women’s mean estradiol (E2) levels on the day they received human chorionic gonadotropin (hCG) were 1812 pmol/L, while progesterone (P4) was 0.59 nmol/L and luteinizing hormone (LH) was 2.94 IU/L. Among the clinical results, 39 specimens had chemical pregnancy after implantation (48%), 33 clinical pregnancies (41%), 30 measurable fetal heartbeats (38%), and 25 live births (31%). There were no significant differences in the physiological values, including the number of eggs retrieved, implanted oocytes, the fertilization rate, and the clinical outcomes among various groups (Table 1).

### 2.2. Sperm miRNA Levels in Men with Teratozoospermia or Asthenozoospermia

**Figure 2:**
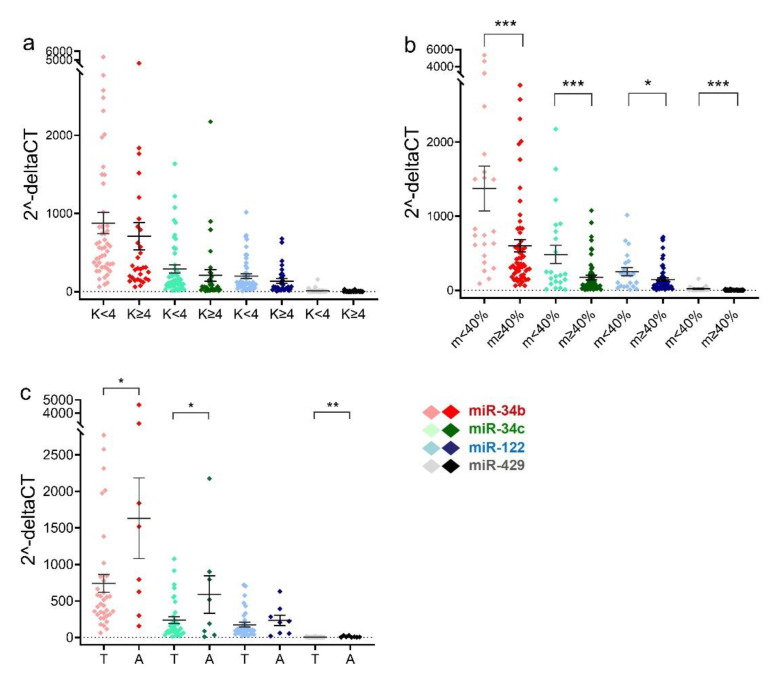
Relative expression levels of miRNAs in spermatozoa with differential morphology and motility. (**a**) Scatter plot showing sperm relative levels of miR-34b/c, miR-122, and miR-429 of each man in the K < 4 and K ≥ 4 groups. Kruger value (K) serves as a criterion for identifying sperm morphology. * *p* < 0.05. K < 4, *n* = 49; K ≥ 4, *n* = 32. (**b**) Scatter plot showing sperm relative levels of miR-34b/c, miR-122, and miR-429 for each individual in motility m < 40% and ≥40 groups. m < 40%, *n* = 22; m ≥ 40%, *n* = 59. * *p* < 0.05, *** *p* < 0.001. (**c**) Differential relative levels of miR-34b/c, miR-122, and miR-429 between spermatozoa from teratozoospermia-only (*n* = 35) and asthenozoospermia-only (*n* = 8) men. The data are shown as mean and SEM. A, asthenozoospermia; T, teratozoospermia. * *p* < 0.05, ** *p* < 0.01.

The relative levels of miR-34b/c, miR-122, and miR-429 in spermatozoa were not significantly different between the teratozoospermia group and the non-teratozoospermia group (Figure 2a). Sperm from asthenozoospermic (<40% motility) men had significantly higher levels of these four miRNAs than non-asthenozoospermic men (*p* = 0.0010, 0.0008, 0.0378, and <0.0002, respectively) (Figure 2b). Comparing teratozoospermic and asthenozoospermic sperm samples, asthenozoospermic sperm showed significantly higher levels of miR-34b/c and miR-429 than teratozoospermic sperm (*p* = 0.0174, 0.0259, and 0.0068, respectively) (Figure 2c).

### 2.3. Comparison of Sperm miRNAs Levels in Different Groups of Fertilization Rate and Early Embryonic Development

**Figure 3:**
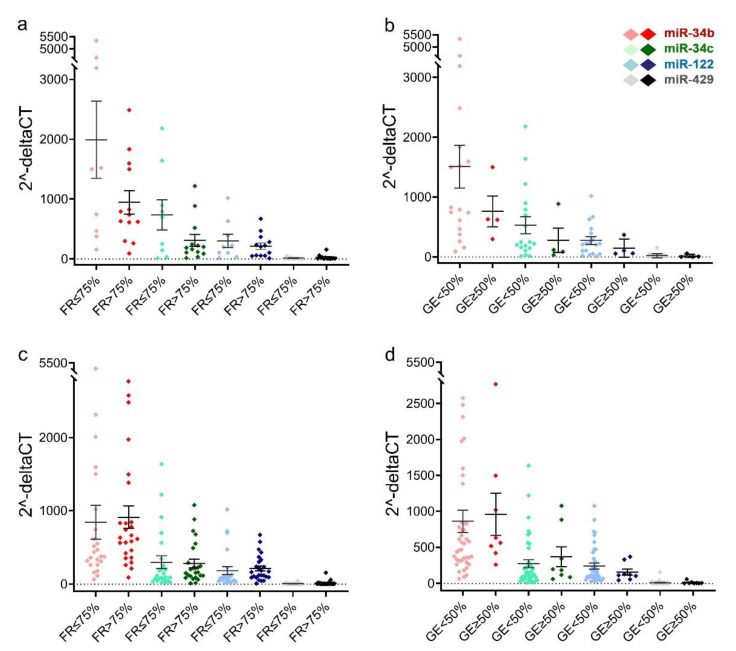
Relative levels of sperm miRNAs in different groups of egg fertilization rates and early embryonic development. Differences in relative levels of miRNAs: (**a**) asthenozoospermia sperm samples between egg fertilization rates >75% and ≤75%, (**b**) asthenozoospermia sperm samples between good-quality embryo rates ≥50% and <50%, (**c**) teratozoospermia sperm samples between egg fertilization rates >75% and ≤75%, and (**d**) teratozoospermia sperm samples between good-quality embryo rates ≥50% and <50%. The data are shown as mean and SEM. FR: fertilization rate; GE: good-quality embryo.

Relative expression levels of miR-34b, miR-34c, miR-122, and miR-429 in asthenozoospermic sperm showed no differences between egg fertilization rates >75% and ≤75% (Figure 3a). Additionally, the groups with a good-quality embryo rate above 50% also did not differ from those with a good-quality embryo rate below 50% for these four miRNAs (Figure 3b).

There were no differences between these four miRNAs in teratozoospermia and egg fertilization rate (Figure 3c). Additionally, the groups with a good-quality embryo rate above 50% still did not differ from those with a good-quality embryo rate below 50% for these four miRNAs (Figure 3d).

### 2.4. Relative Expression Levels of Sperm miRNAs in Different Clinical Outcome Groups after ICSI

**Figure 4:**
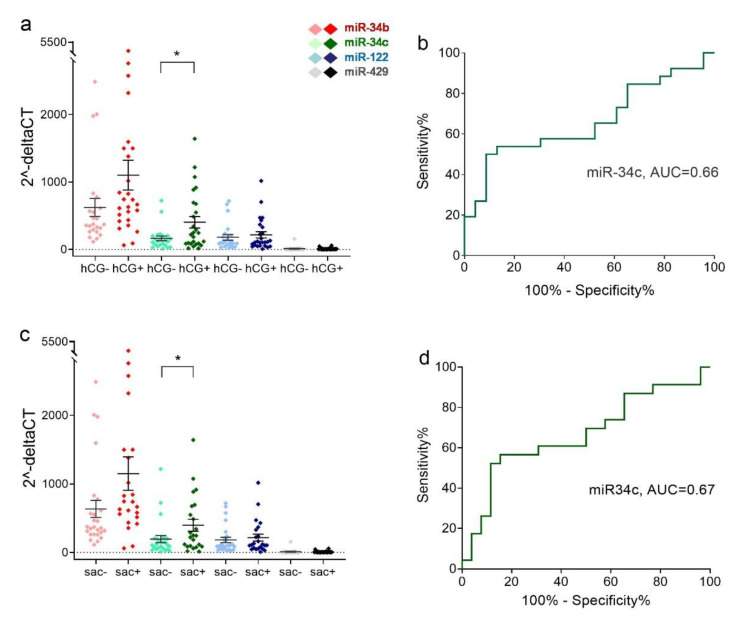
Relative expression levels of miR-34b and miR-34c in spermatozoa of teratozoospermia patients in clinical pregnancy and non-pregnant groups. (**a**) The scatter plot shows microRNA relative levels in each patient with or without urine hCG. (**b**) ROC curve analysis of miR-34c. (**c**) The scatter plot shows microRNA relative levels in each patient with or without a gestational sac under ultrasound detection. (**d**) ROC analysis curve of miR-34b. AUC denotes the area under the curve. The data are shown as mean and SEM. * *p* < 0.05.

In the hCG-positive (hCG+) group, only the miR-34c level was significantly higher in teratozoospermia sperm miRNAs than in the hCG- group (*p* = 0.0157) (Figure 4a). The delta threshold cycle (ΔCt) values were used for statistical analysis. The area under the ROC curve was 0.66 (*p* = 0.0520) (Figure 4b). In the gestational sac-positive (sac+) group, only the miR-34c level was significantly higher in teratozoospermia sperm miRNAs than in the sac- group (*p* = 0.0448) (Figure 4c). The area under the ROC curve was 0.67 (*p* = 0.0451) (Figure 4d). There were no differences in the expression of the four miRNAs between hCG- and hCG+, sac- and sac+ in asthenozoospermia sperm (data not shown).

**Figure 5:**
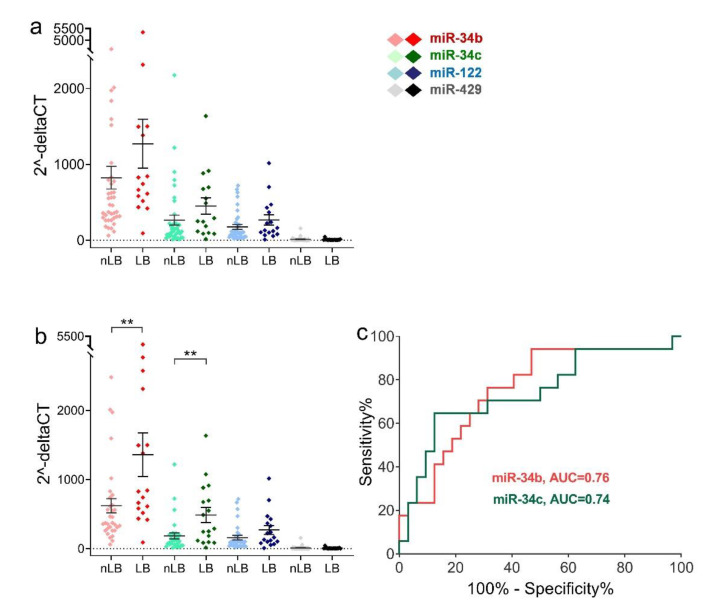
Relative expression levels of miRNAs in male factor sperm and teratozoospermic sperm in live birth and non-live birth groups. (**a**) Scatter plot showing sperm miRNA relative levels for each male factor patient with or without live birth. (**b**) Scatter plot showing sperm miRNA relative levels for each teratozoospermia patient with or without live birth. (**c**) ROC curve analysis of miR-34b/c. The data are shown as mean and SEM. LB, live birth; nLB, non-live birth; AUC, area under the curve. ** *p* < 0.01.

There were no statistical differences in levels of the four miRNAs in male factor sperm (Figure 5a) and asthenozoospermic sperm (not shown) between the live birth group and the non-live birth group. However, the levels of miR-34b and miR-34c in teratozoospermic sperm in the live birth group were significantly higher than those in the non-live birth group (*p* = 0.0084 and 0.0033, respectively) (Figure 5b). For the ability of ROC to predict live birth, the AUCs for miR-34b and miR-34c were 0.76 (95% confidence interval (CI): 0.61–0.90, *p* = 0.0033) and 0.74 (95% CI: 0.59–0.90, *p* = 0.0055), respectively (Figure 5c).

Using ROC analysis to estimate live births, the optimal ∆Ct cutoffs for miR-34b and miR-34c were determined to be 8.630 (sensitivity: 0.9412, specificity: 0.5313) and 7.883 (sensitivity: 0.6471, specificity: 0.8750), respectively.

We then used the miR-34b ∆Ct value of 8.630 as the threshold for reclassifying teratozoospermia specimens. ΔCt value > 8.630 is miR-34b positive, and less than 8.630 is miR-34b negative. As shown in Table 2, there were no significant differences in the fertilization rate and good-quality embryo rate between the two groups. However, the rates of implantation, hCG+, sac+, fetal heartbeat (FHB)+, and live birth were significantly higher in the miR-34b (+) group compared with the miR-34b (−) group. In contrast, while the miscarriage rate was much lower in the miR-34 b (+) group, it did not reach statistical significance.

Next, we grouped the teratozoospermia samples using the miR-34c ΔCt value of 7.883 as a threshold. Compared with miR-34c (−) group, there were no significant differences in the fertilization rate and high-quality embryo rate, but the implantation, hCG+, sac+, FHB+, and live birth rates were significantly increased in the miR-34c (+) group. The miscarriage rate in the miR-34c (+) group was relatively lower, the same trend as that in the miR-34b (+) group (Table 3).

Additionally, we applied the thresholds to all male factor subjects. Higher sperm miR-34b levels showed significantly higher implantation, sac+, fetal heartbeat, and live birth rates (Table S1). Higher sperm miR-34c levels indicated higher implantation, chemical pregnancy, clinical pregnancy, fetal heartbeat, and live birth rates (Table S2). Miscarriage rates were similarly low when sperm had higher levels of these two miRNAs (Tables S1 and S2). If thresholds were used in all ICSI samples in this study, miR-34c showed higher potency than miR-34b in all clinical outcomes (see Tables S3 and S4).

Next, we built two-group multivariate regression models, 1 and 2, to predict the live birth probability for the teratozoospermia samples. Logistic regression analysis included miRNA expression levels and other factors that may affect live birth, including the number of oocytes received, good-quality embryos, number of implanted embryos, days of embryo transfer, female age, and year of infertility. Then, a table of regression coefficients of the equation models was established based on variables (Table S5). The results showed that in addition to the ΔCt values of miR-34b and -34c (*p* = 0.026 and 0.015, respectively), the days of embryo transfer (*p* = 0.017 and 0.016, respectively) in models 1 and 2 were also significantly associated with live birth. These findings suggest that sperm miR-34b and -34c are critical predictors of ICSI clinical outcomes.

For self-validation of logistic regression models, data were examined using the two models to obtain estimated live birth rates. We drew ROC curves based on the predicted and actual values. The miR-34b AUC was 0.887 (95% CI: 0.7855 to 0.9902), diagnostic sensitivity was 76.47%, and specificity was 87.50% (Figure S1a). The miR-34c AUC was 0.9007 (95% CI: 0.7991 to 1.000); the diagnostic sensitivity was 88.24%, and specificity was 90.63% (Figure S1b).

## 3. Discussion

This study found that the differential expression levels of miR-34b and miR-34c in spermatozoa of men with teratozoospermia may be highly correlated with clinical outcomes of ICSI, especially implantation, pregnancy, and live birth rates, suggesting that sperm miR-34b and miR-34c levels could be used as indicators to predict ICSI clinical outcomes. This study was characterized by a relatively higher mean age of both men and women, with an average of 39.11 years for men and 36.94 years for women. A previous study on multiple male factor infertility, mainly oligoasthenoteratozoospermia in younger men, with an average age of 32.85 years in men and 31.14 years in women, found that sperm miR-34c levels were significantly correlated with rates of implantation, pregnancy, and live birth [21]. To date, there are only two research papers on the correlation between sperm miRNA levels and ICSI outcomes, which are studies at different ages. The present study may provide valuable information for middle-aged patients seeking assisted reproduction.

There are limited studies on the levels of the four miRNAs studied in this paper in male factor infertility sperm. Oligozoospermic and asthenozoospermic men have lower sperm miR-34b levels than normozoospermic men [27,32,33]. Sperm miR-34c levels were significantly lower in men with idiopathic infertility than in men with normal semen parameters [21]. The expression level of miR-122 in the sperm of oligozoospermic and asthenozoospermic men was lower than that of normozoospermic men [27,28]. It has also been reported that miR-122 levels were significantly increased in spermatozoa of men with severe and moderate oligoasthenoteratozoospermia compared with normozoospermia [26]. In addition, miR-429 levels increased in asthenozoospermic and oligozoospermic sperm [27,30]. Experiments in bulls found that miR-34 b/c levels were increased in high motile semen, while levels of miR-122 were decreased [29]. We did not attempt to compare the levels of these four miRNAs between teratozoospermic, asthenozoospermic, and normozoospermic sperm but instead focused on correlations with morphology and motility. We found that miR-34b/c, miR-122, and miR-429 levels were significantly elevated in asthenozoospermic men. In addition, the relative expression levels of miR-34 b/c and miR-429 in asthenozoospermic sperm samples were considerably higher than in teratozoospermic sperm samples. These findings suggest that sperm-specific miRNA levels may be associated with sperm motility.

Higher expression levels of miR-34c in sperm may result in good embryo outcomes, as found in humans [21], mice [20], and bovine [34,35]. In our study, we found that asthenozoospermic sperm had higher miR-34c expression than non-asthenozoospermic sperm; however, our patient’s population is relatively older and is the case of ICSI, which means that these men may have unexplained infertility before they come to the hospital for ICSI treatment even if their sperm motility is normal. Thus, it cannot be seen that asthenozoospermic sperm have higher expression of miR-34c than normal sperm in this study. Further studies are needed to verify relative expression levels of miR-34c in asthenozoospermia and normozoospermic sperm.

Sperm-derived miRNAs may affect fertilization and early embryonic development before implantation [20,36,37]. A previous study showed that sperm with high expression of miR-191 had higher fertilization rates [38]. However, no correlation was found between miR-34b/c levels and fertilization rates [21]. We found that the sperm miRNAs studied here were not associated with >75% egg fertilization rates. Cui et al. found that an increased expression level of miR-34c in spermatozoa might be associated with preimplantation embryonic development [21]. Additionally, our study showed that sperm miR-34b/c, miR-122, and miR-429 were not related to early embryonic development. However, when miR-34b/c is higher than a certain threshold, the implantation and pregnancy rates increase significantly, and the miscarriage rate decreases, indicating that the quality of early embryos should be better. Theoretically, miR-34b/c could have specific effects on early embryonic development. A previous study found that sperm miR-34c may affect early embryonic development [21]. Although our results showed a trend towards an effect, they did not reach a statistically significant difference and may require more cohorts to confirm in the future.

Mouse studies have shown that sperm miR-34c is essential for the first cell division of early embryos [20]; however, another paper reported that it is not required for the first cleavage division [37]. In human studies, sperm miR-34c levels have been found to correlate with early embryo development [21], but another paper reported no correlation [38]. For bovines, one study showed that microRNA-34c expression in donor cells affects early development of somatic cell nuclear transfer embryos [35]. Another study suggested that miR-34 may be required in bovine gametes developing both sexes and embryos [34]. Additionally, also, the expression of miR-34c-3p in bull spermatozoa correlates with fertility rates [39].

We also found a significant correlation between sperm miR-34b/c above threshold levels and ICSI outcomes in teratozoospermia patients (Table 2 and Table 3). These outcomes include implantation, pregnancy, and live birth rates. The miscarriage rate, although not statistically significant, is relatively low. If the samples included patients with asthenozoospermia, miR-34-b/c still showed a significant correlation (Tables S1 and S2). When non-male factor samples were also included in the classification, only miR-34c revealed effective pregnancy and live birth rates (Table S3).

In screening for live births, the results in Table 2 and Table 3 show that miR-34c is more effective than miR-34b (miR-34c+ vs. miR-34b+, 73.33% vs. 51.61%, respectively). The positive likelihood ratios for miR-34c and miR-34b are 5.12 and 2.00, respectively. In addition, the samples belonging to the miR-34c+ group also belonged to the miR-34b+ samples; this situation is present in teratozoospermia samples (*n* = 49) and all ICSI samples (*n* = 81). The results of the multivariate logistic analysis also found that miR-34c had a more significant effect on live birth than miR-34b under various variables (AUC, 0.9007 vs. 0.8879, respectively).

A previous study also found that sperm miR-34c levels were a good indicator of clinical outcomes in male infertile patients. Most had multiple sperm disorders and were treated with ICSI [21]. A study of IVF of normal sperm samples and the eggs of female partners with defective fallopian tubes found that sperm miR-34c levels were significantly correlated with clinical outcomes of IVF in the presence of normal sperm and eggs [22]. These findings and our study results suggest sperm miR-34c may be a promising biomarker for ICSI and IVF outcomes.

## 4. Materials and Methods

### 4.1. Sperm Sample Collection and Processing

Sperm samples were obtained from male partners of couples who underwent ICSI for infertility through assisted reproductive technology at MacKay Memorial Hospital Reproductive Medicine Center. Screening criteria for ICSI treatment include male factor infertility, poor fertilization rate in the previous IVF cycle, unexplained infertility, or patients with frozen and thawed eggs. Exclusion criteria are azoospermia and samples with a total sperm count of less than 5 × 10^6^/mL after washing. Samples were obtained by masturbation after participants had abstained from sex for 3 days. After liquefying the semen samples for 30 min at room temperature on the day of ICSI treatments, all semen samples were analyzed for the main semen parameters according to the World Health Organization 2010 guidelines [40]. There are two test subgroups of the experiment: teratozoospermia is defined according to Kruger’s Strict Criteria (Kruger, K < 4%), while asthenozoospermia is defined as progressive motility + non-progressive motility <40%. The remaining sperm after ICSI is temporarily stored in a fresh Quinn’s Advantage Fertilization medium at room temperature. After confirmation of oocyte fertilization, sperm samples were counted and held at −80 °C to quantify miRNA levels. The study was approved by the MacKay Memorial Hospital’s Institutional Review Board (approval number: 17MMHIS060), and informed consent was obtained from each participant.

### 4.2. Assisted Reproductive Technology

Controlled ovarian stimulation was initiated on the third day of the menstrual cycle for three consecutive days following the baseline pelvic ultrasound scan. All subjects received a fixed starting dose of 300 IU of recombinant follicle-stimulation hormone (r-FSH) (Gonal-F; Merck Serono Biopharma). Subsequent daily r-FSH doses were then adjusted based on ovarian response. A gonadotropin-releasing hormone antagonist (Cetrotide; Merck Serono Biopharma) was added to the stimulation protocol when at least one follicle reached 14 mm in diameter. We then triggered final oocyte maturation using 6500 IU of recombinant hCG (Ovidrel; Merck Serono Biopharma) or 6500 IU of recombinant hCG and 0.2 mg of triptorelin (Decapeptyl; Ferring Pharmaceuticals) when at least two leading follicles reached 18 mm in diameter. The choice of triggering method was based on the attending physician’s discretion. Oocyte retrievals were performed under transvaginal ultrasound guidance, 35 to 36 h post triggering. Standard ICSI was performed. According to embryo development, it is transferred between day 2 and day 5 after oocyte retrieval.

### 4.3. Fertilization and Determination of Clinical Outcomes

The retrieved mature oocytes, the metaphase II oocytes, were fertilized by ICSI. After 16–18 h, the number of pronuclei in the oocyte was checked, and the presence of two pronuclei was the criterion for successful fertilization. Fertilized eggs were cultured in the global total medium (LifeGlobal) at 37 °C and 6% CO_2_, and the embryo quality was analyzed on the second day after fertilization. High-quality embryos were defined as embryos with 4 uniformly sized dividing cells on day 2 or embryos containing 8–10 uniformly sized cells with less than 10% fragmentation on day 3. This study denoted a good-quality embryo as more than one high-quality embryo on day 2 or 3. According to the conditions of the embryos and patients, we selected 1–3 embryos with the best grades for transfer (day 2–day 5) and tracked the clinical pregnancy outcomes after embryo transfer. Two weeks after transfer, serum β-hCG was taken to confirm the biochemical pregnancy. Clinical pregnancy was defined as the presence of an intrauterine gestational sac on ultrasound 4 weeks after transfer, and fetal heartbeat was detected by transvaginal ultrasound 5 weeks after transfer. The implantation rate was defined as the number of gestational sacs observed on ultrasound screening divided by the number of embryos transferred. Live birth was defined as live births of an infant equal to or greater than one living fetus at the gestational age >20 weeks.

### 4.4. TaqMan MicroRNA Quantitative Polymerase Chain Reaction

Sperm concentration was estimated using a Makler counting chamber under a microscope with 400 times magnification. Approximately 5 × 10^6^ sperm was precipitated by centrifuge at 15,000 rpm for 10 min at room temperature, and 700 μL QIAzol lysis reagent (Qiagen, #217004) was added to the precipitated sperm sample and shaken to mix well. Total RNA was extracted using the miRNA Isolation Kit (Qiagen, #217004). TaqMan microRNA Reverse Transcription Kit (Thermo Fisher Scientific, #PN4366597) was used to reverse-transcribe microRNAs to their complementary DNAs (cDNAs) using target-specific stem-loop reverse transcription primers for miR-34b (#002102), miR-34c (#000428), miR-122 (#002245), miR-429 (#001024), and RNU6B (#001093). Real-time quantitative polymerase chain reaction (qPCR) was conducted in a total volume of 20 μL, containing the above-described cDNAs, the corresponding miRNA probes and primers, and the TaqMan Universal Master Mix II (#4440038). All reactions were performed in triplicate and ran on the 7500 Fast Real-Time PCR System (Applied Biosystems) under the following conditions: 95 °C for 10 min, followed by 40 cycles at 95 °C for 15 s and 60 °C for 1 min. The Ct value was determined automatically by 7500 Fast System software version 2.0.1, and Ct values <35 were included in the analysis. microRNA ΔCt was calculated by subtracting the Ct value of miRNAs from the Ct value of RNU6B. Relative quantification of microRNA expression was calculated using the 2^−ΔCt^ method [41]. The value of microRNA ΔCt was used to analyze the association of sperm microRNAs with ICSI outcomes statistically.

### 4.5. Statistical Analysis

Data are presented as mean ± standard error of the mean. Between-group variables were analyzed using independent-samples *t*-tests and one-way ANOVA, as appropriate. Fisher’s exact test was used to compare ratios between groups. Factors associated with live birth were identified using logistic regression analysis. Receiver operating characteristic curves were used to establish cutoff thresholds and diagnostic values. Analyses were performed using GraphPad Prism version 6 (GraphPad, San Diego, CA, USA) and SPSS software version 26 (IBM Corp., Armonk, NY, USA). A *p*-value < 0.05 was considered statistically significant.

## 5. Conclusions

The differential expression levels of miR-34b and miR-34c in the sperm of teratozoospermic men above a certain threshold have significantly higher implantation, pregnancy, and live birth rates, suggesting that miR-34b and miR34c may be promising predictors of ICSI outcomes.

## Figures and Tables

**Table 1:** Patient characteristics.

Items	All	nMF	MF	Terato.	Astheno.	*p*-Value
N	81	24	57	49	22	NS
Women’s age (y)	36.94 ± 4	37.08 ± 4	36.88 ± 4	36.78 ± 4	37.14 ± 4	NS
Men’s age (y)	39.11 ± 6	38.38 ± 6	39.42 ± 5	39.31 ± 5	40.64 ± 5	NS
Infertility year	3.48 ± 3	3.42 ± 3	3.51 ± 3	3.61 ± 3	3.59 ± 2	NS
Oocyte retrieved no.	10.43 ± 6	9.96 ± 4	10.63 ± 6	11.00 ± 7	10.05 ± 5	NS
Good embryo no.	1.35 ± 2	1.04 ± 1	1.47 ± 6	1.43 ± 2	1.41 ± 2	NS
Transferred embryo no.	2.36 ± 1	2.50 ± 1	2.30 ± 1	2.31 ± 1	2.23 ± 1	NS
E2 on hCG day (pmol/L) ^a^	1812 ± 991	1634 ± 863	1851 ± 1060	1875 ± 1113	1822 ± 938	NS
P4 on hCG day (nmol/L) ^a^	0.59 ± 0.36	0.54 ± 0.23	0.61 ± 0.40	0.62 ± 0.41	0.69 ± 0.54	NS
LH on hCG day (IU/L) ^a^	2.94 ± 6	4.13 ± 9	2.33 ± 2	2.14 ± 1.96	3.18 ± 2	NS

^a^ Does not include egg freezing and thawing samples.

**Table 2:** Comparison of sperm miR-34b levels and ICSI outcomes in men with teratozoospermia.

ICSI Outcomes	miR-34b(−) ^1^	miR-34b(+) ^2^	*p*-Value
Cycle (*n* = 49)	18	31	
Normal fertilization rate (per MII, %)	74.01%	82.42%	0.0696
More than 50% good-quality embryo rate (%)	5.56%	22.58%	0.2293
Implantation rate (per embryo, %)	5.50%	43.52%	0.0003
hCG+ rate (per embryo transfer, %)	27.78%	67.74%	0.0090
Sac^+^ rate (per embryo transfer, %)	16.67%	64.52%	0.0025
FHB^+^ rate (per embryo transfer, %)	16.67%	58.06%	0.0069
Live birth rate (per embryo transfer, %)	5.56%	51.61%	0.0014
Miscarriages rate (%)	40.00%	14.28%	0.2357

^1^ miR-34b (−), ΔCt ≤ 8.630; ^2^ miR-34b (+), ΔCt > 8.630.

**Table 3:** Comparison of sperm miR-34c levels and ICSI outcomes in men with teratozoospermia.

ICSI Outcomes	miR-34c (−) ^1^	miR-34c(+) ^2^	*p*-Value
Cycle (*n* = 49)	34	15	
Normal fertilization rate (per MII, %)	78.24%	81.87%	0.4582
More than 50% good-quality embryo rate (%)	14.71%	20.00%	0.6869
Implantation rate (per embryo, %)	20.56%	49.93%	0.0092
hCG+ rate (per embryo transfer, %)	38.24%	86.67%	0.0021
Sac^+^ rate (per embryo transfer, %)	32.35%	80.00%	0.0043
FHB^+^ rate (per embryo transfer, %)	26.47%	80.00%	0.0012
Live birth rate (per embryo transfer, %)	17.65%	73.33%	0.0003
Miscarriages rate (%)	36.35%	8.33%	0.1550

^1^ miR-34c (−), ΔCt ≤ 7.883; ^2^ miR-34c (+), ΔCt > 7.883.

## Data Availability

The data presented in this study are available on request from the corresponding author.

## Supplementary Materials

The following are available online at https://www.mdpi.com/article/10.3390/ijms232012381/s1.
