# Supplementary material for: Correlation between Sperm Micro Ribonucleic Acid-34b and -34c Levels and Clinical Outcomes of Intracytoplasmic Sperm Injection in Men with Male Factor Infertility
Source: Int J Mol Sci. 2022 Oct 16;23(20):12381. doi: 10.3390/ijms232012381 (PMC9604024; doi:10.3390/ijms232012381)
Supplement: Supplementary file 1 [file ijms-23-12381-s001.zip › ijms-1947700-supplementary.pdf]

Supplemental Materials

Table S1. Comparison of sperm miR-34b levels and ICSI outcomes in male factor samples.

ICSI outcomes	miR-34b(-)[1]	miR-34b(+)[2]	*p*-Value
Cycle (n = 57)	20	37	
Normal fertilization rate (per MII, %)	73.60%	81.49%	0.0738
More than 50% good-quality embryos (%)	10.00%	21.62%	0.4674
Implantation rate (per embryo, %)	4.95%	36.46%	0.0011
hCG+ rate (per embryo transfer, %)	30.00%	56.00%	0.0943
Sac$^+$ rate (per embryo transfer, %)	15.00%	54.05%	0.0049
FHB$^+$ rate (per embryo transfer, %)	15.00%	48.65%	0.0201
Live birth rate (per embryo transfer, %)	5.00%	43.24%	0.0024
Miscarriages rate (%)	50.00%	14.00%	0.1009

[1] miR-34b(-), ΔCt ≤ 8.630; [2] miR-34b(+), ΔCt > 8.630

Table S2. Comparison of sperm miR-34c levels and ICSI outcomes in male factor samples.

ICSI outcomes	miR-34c(-)[1]	miR-34c(+)[2]	*p*-Value
Cycle (n = 57)	38	19	
Normal fertilization rate (per MII, %)	78.47%	79.21%	0.8708
More than 50% good-quality embryos (%)	18.42%	15.79%	0.9999
Implantation rate (per embryo, %)	18.39%	39.42%	0.0360
hCG+ rate (per embryo transfer, %)	36.84%	68.42%	0.0475
sac+ rate (per embryo transfer, %)	28.95%	63.16%	0.0214
FHB+ rate (per embryo transfer, %)	23.68%	63.16%	0.0077
Live birth rate (per embryo transfer, %)	15.79%	57.89%	0.0019
Miscarriages rate (%)	45.45%	8.33%	0.0686

[1] miR-34c(-), ΔCt ≤ 7.883; [2] miR-34c(+), ΔCt > 7.883

Table S3. Comparison of sperm miR-34b levels and ICSI outcomes in all ICSI samples.

ICSI outcomes	miR-34b(-)[1]	miR-34b(+)[2]	*p*-Value
Cycle (n = 81)	37	44	
Normal fertilization rate (per MII, %)	73.78%	78.61%	0.2037
More than 50% good-quality embryos (%)	16.22%	20.45%	0.7759
Implantation rate (per embryo, %)	22.27%	32.93%	0.2161
hCG+ rate (per embryo transfer, %)	46.00%	50.00%	0.8242
Sac$^+$ rate (per embryo transfer, %)	32.43%	47.73%	0.1810
FHB$^+$ rate (per embryo transfer, %)	29.73%	43.18%	0.0323
Live birth rate (per embryo transfer, %)	21.62%	38.64%	0.1470
Miscarriages rate (%)	35.00%	14.00%	0.2431

[1] miR-34b(-), ΔCt ≤ 8.630; [2] miR-34b(+), ΔCt > 8.630

Table S4. Comparison of sperm miR-34c levels and ICSI outcomes in all ICSI samples.

ICSI outcomes	miR-34c(-)[1]	miR-34c(+)[2]	*p*-Value
Cycle (n = 81)	59	22	
Normal fertilization rate (per MII, %)	77.91%	75.85%	0.6296
More than 50% good-quality embryos (%)	20.34%	13.64%	0.7486
Implantation rate (per embryo, %)	24.14%	38.59%	0.1334
hCG+ rate (per embryo transfer, %)	42.00%	64.00%	0.1331
sac+ rate (per embryo transfer, %)	33.90%	59.09%	0.0466
FHB+ rate (per embryo transfer, %)	28.81%	59.09%	0.0191
Live birth rate (per embryo transfer, %)	22.03%	54.55%	0.0072
Miscarriages rate (%)	35.00%	8.00%	0.1189

[1] miR-34c(-), ΔCt ≤ 7.883; [2] miR-34c(+), ΔCt > 7.883

Table S5. Regression coefficients of models 1 and 2 for the teratozoospermia samples.

Factors	OR	95% CI	OR	95% CI
	Model 1		Model 2	
ΔCt value of miR-34b	2.235*	1.099-4.543		
ΔCt value of miR-34c			2.032 *	1.149-3.593
Numbers of oocytes received	0.871	0.741-1.023	0.847	0.715-1.004
Numbers of good-quality embryos	0.707	0.406-1.233	0.694	0.397-1.213
Numbers of implanted embryos	0.634	0.157-2.567	0.758	0.164-3.503
Embryo transfer days	3.624*	1.256-10.452	3.867 *	1.287-11.616
Woman's age (y)	0.923	0.665-1.282	0.913	0.645-1.294
Infertile year	1.286	0.882-1.874	1.212	0.822-1.787

* $p < 0.05$

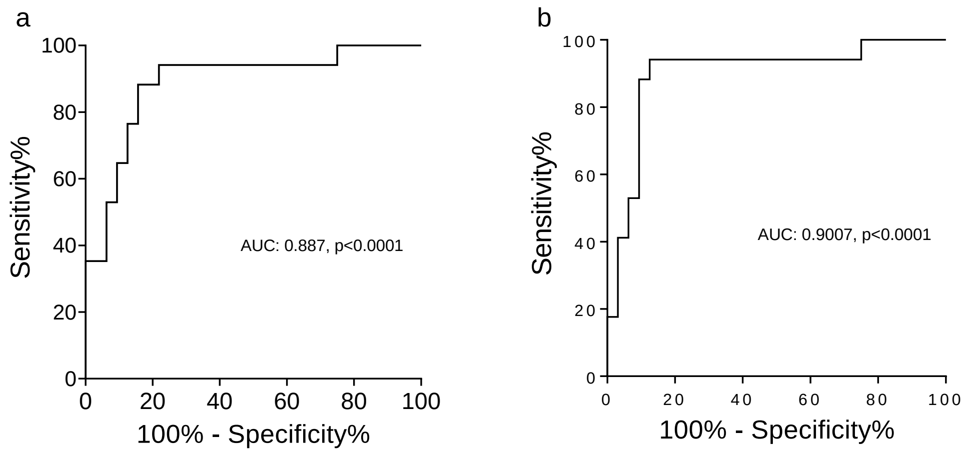

Figure S1. ROC curve analysis of miR-34b/c. (a) miR-34b, model 1. (b) miR-34c, model 2.
